# EEG to Primary Rewards: Predictive Utility and Malleability by Brain Stimulation

**DOI:** 10.1371/journal.pone.0165646

**Published:** 2016-11-30

**Authors:** Nicole Prause, Greg J. Siegle, Choi Deblieck, Allan Wu, Marco Iacoboni

**Affiliations:** 1 Department of Psychiatry; University of California;Los Angeles, CA; 2 Western Psychiatric Institute and Clinic, University of Pittsburgh, Pittsburgh, PA; 3 Ahmanson-Lovelace Brain Mapping Center, University of California, Los Angeles, Los Angeles, CA; University Medical Center Goettingen, GERMANY

## Abstract

Theta burst stimulation (TBS) is thought to affect reward processing mechanisms, which may increase and decrease reward sensitivity. To test the ability of TBS to modulate response to strong primary rewards, participants hypersensitive to primary rewards were recruited. Twenty men and women with at least two opposite-sex, sexual partners in the last year received two forms of TBS. Stimulations were randomized to avoid order effects and separated by 2 hours to reduce carryover. The two TBS forms have been demonstrated to inhibit (continuous) or excite (intermittent) the left dorsolateral prefrontal cortex using different pulse patterns, which links to brain areas associated with reward conditioning. After each TBS, participants completed tasks assessing their reward responsiveness to monetary and sexual rewards. Electroencephalography (EEG) was recorded. They also reported their number of orgasms in the weekend following stimulation. This signal was malleable by TBS, where excitatory TBS resulted in lower EEG alpha relative to inhibitory TBS to primary rewards. EEG responses to sexual rewards in the lab (following both forms of TBS) predicted the number of orgasms experienced over the forthcoming weekend. TBS may be useful in modifying hypersensitivity or hyposensitivity to primary rewards that predict sexual behaviors. Since TBS altered the anticipation of a sexual reward, TBS may offer a novel treatment for sexual desire problems.

## Introduction

Reward processing abnormalities, particularly abnormally high reward sensitivity, appears key to many psychopathologies from addictions to mood disorders [1]. Individual differences in sensitivity to novelty and rewards are associated with impulsivity [2] and general risk behaviors [3]. In turn, these are linked to specific risk behaviors. Sexual risks are particularly important due to their potentially major negative consequences including disease, pregnancy, and social consequences. Impulsive responding has been associated with risky sexual behaviors including unprotected sex [4], sex with strangers and inconsistent condom use [5] and more partners [6]. One goal of the present study was to use neural responses to sexual cues to predict future potentially risky sexual behaviors.

The primary goal was to examine the extent to which these neural responses could be directly modulated. Transcranial magnetic stimulation (TMS) has specifically been used to address problems of low reward responsiveness, especially anhedonia in depression [7]. TMS also is used to decrease cravings for tobacco [8], cocaine [9], alcohol [10], and food [11], suggesting TMS may normalize reward sensitivity. TMS is thought to depolarize neurons under the coil by magnetic pulses. Some TMS methods can disrupt functions in distal networks by affecting connectivity, which is consistent with inducing plasticity [12]. TMS to L-DLPFC increased dopamine in hippocampus [13], nucleus accumbens [14], putamen [15] and striatum [16]. Dopamine appears to have several roles in learning [17] and anticipating rewards [18]. Roles include predicting the likelihood of reward, and generating emotional responses prior to a potentially rewarding stimulus [19]. Prefrontal dopamine further appears important for executive processes of control [20]. Thus, TMS could affect any of these processes. Relatedly, TMS also speeds the learning of associations between behaviors and rewards [21]. The present study used a form of TMS, theta burst stimulation (TBS) in those engaging in risky sexual behaviors. If the reward prediction of sex can be downregulated, decreased anticipation might improve sexual decision making. This change also may have implications for sexual compulsivity.

TBS has demonstrated an effect of reducing impulsive decisions [22] likely through plasticity [23] induced in a distributed cortical network [24]. The pattern of TBS delivered has been demonstrated to increase or decrease activity in motor areas [25]. Intermittent TBS (iTBS; 2 trains of TBS repeated every 10 s to 600 pulses), increased cortical activity. Conversely, continuous TBS (cTBS; 40 s uninterrupted to 600 pulses), decreased cortical excitability [26]. These effects appear to last for about 60 minutes after stimulation [27, 28]. Excitability of motor and DLPFC cortices are positively related [29] and DLFPC activity also is inhibited by cTBS [30].

cTBS might alter sexual responsiveness by enhancing sexual conditioning. cTBS to L-DLPFC spreads activation widely in the resting brain, including through fronto-parietal, and especially cingulo-opercular, networks [31]. L-DLPFC specifically has been functionally linked with the dorsal anterior cingulate cortex [32], the activity of which appears important in upregulating response to sexual cues [33]. This major functional network [34] is thought to maintain attentional set in learning [35, 36]. It also decreased dopamine release, such as in the ipsilateral caudate-anterior putamen and contralateral caudate nucleus [37]. cTBS also increased approach/reward learning [38]. iTBS, on the other hand, could increase the person’s ability to regulate emotions, including sexual emotions. Thus, both cTBS and iTBS have the potential to alter sexual responsiveness via different mechanisms. Since alpha suppression indicates greater engagement, the stimulation method that results in greater alpha suppression during reward (anticipation and receipt) is interpreted as greater sexual responsiveness.

Alpha band activity in EEG was studied due to its associations with rewards and engagement. Alpha represents a relatively low-frequency component of the EEG. Greater power in this spectrum is commonly thought to reflect cortical idling or, more colloquially, a “relaxed wakefulness” [39]. For example, alpha is lower when viewing motion in films as compared to still images [40]. Alpha is thought to emerge from synchronous firing in thalamo-cortical and cortical systems [41], although large swaths of limbic and other system activity also have been related to alpha power [42]. Higher alpha was associated with food rewards in animals [43] and game play (in ventral striatum) in humans [44], and dysynchrony reflected reduced inhibition [45]. Alpha indices vary in both reward hyposensitive [46] and hyper-sensitive (e.g., impulsive, [47]) individuals. This includes acute responses to rewards [48], including sexual rewards [49]. Here, alpha suppression in response to rewards is interpreted as evidence of increased engagement with the sexual cue.

In sum, the present study examined the ability of TMS to alter these neural responses to sexual rewards. Further, the ability of these neural responses to predict future sexual behaviors was tested to examine this aspect of their validity.

## Material and methods

### Recruitment

Participation was solicited using a single posting on Craigslist, which has been used to recruit men engaging in risky sex [50, 51]. The advertisement stated that brain stimulation was being tested to help individuals gain control of their sexual arousal; it did not describe risk behavior requirements to prevent falsification. In the thirty minutes that the posting was live, over 200 contacts were made to the laboratory via email or phone.

Participants were required to be age 18 to 55, identify as born male or female, deny major disease, injury or surgery affecting the genitalia, brain, or spinal cord, deny alcohol or drug abuse in the last week, not be restricted from sexual exertion due cardiovascular risks as assessed by a physician, normal or corrected-to-normal vision and hearing, interest in sex with those of the opposite sex, meet safety requirements for brain stimulation (e.g., no history of seizures, reviewed by [52, 53]). They also must have had at least two different intercourse (vaginal or anal) partners in the last year consistent with being sexually active. No homosexual men or women participated. Partner count is commonly used as an index of risk [54], and those with two or more partners have more sexually transmitted infections [55], elevations in other risky behaviors [56], and are associated with greater sexual compulsivity [57]. This cutoff also has been associated with greater sensitivity to sexual rewards [6].

Twenty-six individuals were screened by the order of call received. These included 16 men and 10 women. Six did not qualify: Five reported too few sexual partners in the last year. One was over the upper age limit. No one declined to participate on hearing the protocol described, which included a description of the genital vibrator used in the study (see below).

### Questionnaires

Participants completed a series of questionnaires. Initial questions included basic demographic information, such as age, education, and relationship status. These characterized their sexual feelings and behaviors [58], the valence of external rewards and punishments by the Sensitivity to Punishment and Sensitivity to Reward Questionnaire [2], Behavioral Inhibition and Activation [59], and Sensation Seeking [60]. To help characterize interest in potential development of TBS as an intervention for sexual responsiveness, participants also were asked to rate to what extent (1) they personally would want to receive more brain stimulation and (2) whether someone they knew would benefit from a brain stimulation approach to reducing sexual responsiveness.

One questionnaire was developed to assess sexual behaviors that occurred over the weekend following the TBS session. Participants indicated for each of Friday, Saturday, and Sunday, how many people they had intercourse with, how many of these individuals were new sexual partners, whether any sexual partner was non-consensual, whether alcohol or other recreational substance preceded sex, and how many orgasms they had each day. This is consistent with a method of “Total sexual outlet” [61] used to include both solitary and partnered sexual behaviors. Consistent with a previous attempt to predict future sexual behaviors [62], these participants did not report enough new partners to allow robust prediction. Thus, the total orgasm count (partnered or non-partnered) reported over the weekend was used for prediction, the occurrence of which may vary with risk level [63]. Participants were not aware of the content of this questionnaire until they accessed the questionnaire after the weekend being assessed had passed.

Participants also completed questionnaires assessing their depression, anxiety, sexual functioning, sexual desire, sexual compulsivity, vibrator use, personality, and mood. Data presented here are limited to the specific hypotheses tested.

### Monetary/Vibratory Incentive Delay (MID/VID)

Two tasks were used to assess shifts in responsiveness to rewards between iTBS and cTBS. These included secondary (monetary) and primary (sexual) rewards. Sexual reward was used as it is specific to the risk-domain under study. Sexual reward is also a primary reward domain that engages brain function similarly to other rewards [64]. More than sports, money, affiliation, and humor, sexual stimuli modulate startle [65, 66], BOLD [67, 68], event-related brain potentials [69, 70], respiration [71], and dopaminergic PET [72]. Reward structures are clearly implicated in sexual anticipation through receipt [73]. Dopamine agonists increase sex behaviors [74] and enhance sexual response [75].

**Fig 1:**
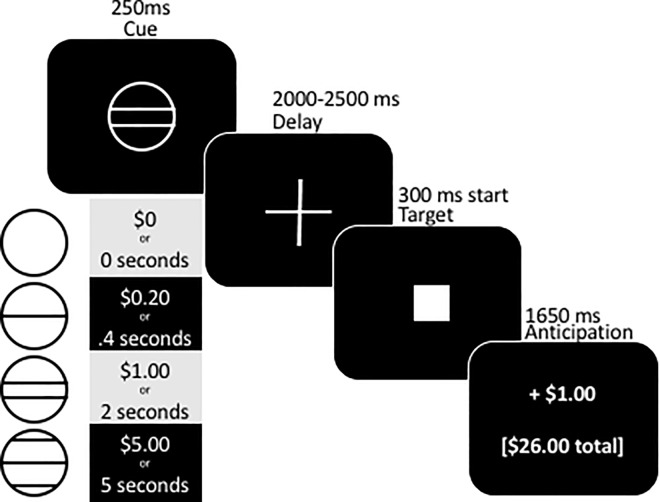
Monetary and Vibratory Incentive Delay (MID/VID) task and reward schedule.

A modified monetary incentive delay [MID, 76] task was used to test responsivity to these secondary rewards. Participants view a shape cue that indicates the magnitude of reward possible (see Fig 1). A solid square appears briefly. If they are able to press a button while the square remains on the screen, they win that trial. The game titrates to achieve a particular win proportion (about 66%), although this is not disclosed to the participant. Initial target duration was set at 300 ms. This task also permits separation of reward anticipation and receipt, since the reward is separated by a time delay from the response [77].

Reward anticipation in the MID is associated with striatal activity [76, 78]. Reward receipt is associated with increased activity in ventromedial frontal cortex [77, 79]. This responsiveness in fMRI studies appears stable over 2.5 years [79]. Also, nucleus accumbens activity during reward anticipation is associated with greater positive arousal (e.g., behavioral activation, extraversion, etc., [79]). The MID task also provokes dopamine activity in posterior caudate [80] and is sensitive to dopamine manipulations [81]. Performance is affected in populations with reward deficits, including schizophrenia [82] and depression [83, 84], and reward hypersensitivity, such as impulsivity problems (e.g., alcoholics as in [85]).

The same task was repeated with genital vibratory reinforcement (VID). Money in the original MID increases logarithmically, so vibratory reinforcement also was logarithmic in duration (see Fig 1). Specifically, the reward magnitude of the genital vibratory stimulation was varied by offering stimulation from .5 to 5 seconds. The MID and VID tasks each took 11.5 minutes each to complete. Both tasks were presented on a 1280 X 1024 LCD monitor at 90° visual angle using Eprime (Psychology Software Tools, Inc., Pittsburgh, PA) with 75 Hz refresh and 32-bit color depth.

### Vibrator parameters

Vibrators offer standardization of stimulation level, evoke stronger sexual response than erotic films, are effective for men [86] and women [87], reduce variability due to third variables (e.g., hand grip strength), and allow inclusion of different sexual orientations (cp., sex films). Genital vibratory stimulation is commonly used for sexual stimulation in research [86, 88–98]. The vibrator is placed on areas self-reported to best provoke the sexual response in men (under the penile glans, [99]) and women (over the clitoral hood [100]). Commercial genital stimulation devices vary widely in their motor characteristics, and testing pointed to the selection of higher displacement devices for research purposes [101].

The vibrator used in this study was a “Magic Wand” (Hitachi) commercially-available vibrator. An external potentiometer permitted participants to select any intensity up to the maximum output of the device. This was binned into 9 equal setting to allow quantification of the intensity. At a setting of “1” no vibration was occurring; at a setting of “9” the maximum setting on the device [101] was used. Maximum stimulation included oscillations at 110 Hz with 40 μm displacement.

Attachments were included as appropriate to the gender of the participant, identified at screening. Participants placed the device themselves after instruction from the experimenter (NP). For females, an oblong extension (the Miracle Massager Accessory; California Exotics) was attached and placed over the clitoral hood (not inserted into the vagina). This area is reported by women to be the area that was easiest to stimulate an orgasm [100]. For men, a sheath that surrounds the penis, the Hummingbird (Love Products) was attached. While men generally indicate greater orgasm sensitivity at the glans [99], the flaccid penis is generally too short to distinguish glans and shaft in the placement of a wide device. Thus, men were instructed only to insert their penis into the attachment.

Given individual differences in genital physiology and stimulation preferences, participants were asked to set the intensity of the vibrator intensity. They were asked to select an intensity that would be “the most pleasurable for up to five seconds”, which is the longest stimulation possible in the VID. Their setting was recorded. No participant reported problems identifying a setting. Participants were offered the opportunity to change the setting for the second testing. All declined.

### Electroencephalography

An EEG robust to movement was desirable. Thus, EEG data were collected using an Emotiv Epoc headset with Emotiv’s TestBench software (Emotiv Systems Inc., San Francisco, CA, USA). This wireless 14-channel headset produces EEG similar to wired headsets [102]. 50k**Ω** impedance estimates are used to indicate sufficient sensor contact **(**G. Mackellar, personal communication, July 21, 2014). EEG channels conform to international 10–20 sites AF3, F7, F3, FC5, T7, P7, O1, O2, P8, T8, FC6, F4, F8, and AF4 were sampled at 128 Hz. The Emotiv headset has been used to demonstrate localization of linguistic processes [103]. Those with clear evoked response potentials in wired (Neuroscan) caps also had clear auditory evoked potentials from Emotiv headsets [104].

### EEG Preprocessing

EEG data were preprocessed with a combination of EEGlab [105], Fieldtrip [106], and custom Matlab (Mathworks 2014) routines. The hardware included a 45 Hz low pass filter. Preprocessing included several steps. The first steps take advantage of the unique features of the Emotiv, which include a mounted accelerometer.1) Accelerometer data were combined $\sqrt{\theta^{2}+\phi^{2}}$, smoothed (1000 point flat kernel), and both raw and smoothed accelerometer data were regressed out of the raw EEG to remove head movement. Removing gross artifacts in the first step is similar to procedures to remove eye movement in EEG [107] and head movements in fMRI [108]. 2) Independent Components Analysis (ICA) using EEGlab’s “runica” function was conducted and components with very high variance (standard deviation > 75^th^ percentile + IQR) or systematically high values (mean < 75^th^ percentile + IQR) were removed as indicating artifact. 3) Bad channels were defined as high variance (standard deviation >75^th^ percentile + 2IQR) or low variance (standard deviation <25^th^ percentile-2IQR) and interpolated from the surrounding channels. 4) Periods of signal loss (see Emotiv above) and outliers (identified as data >100sd from the timeseries mean) were replaced with regression prediction of the two more adjacent unaffected channels prior to transformations into the frequency domain (e.g., [109]). Data were substituted from adjacent channels rather than using more common techniques such as linear interpolation to preserve frequency domain characteristics. 5) Remaining time-frequency domain outliers (>1.5*IQR from Tukey Hinges, 25th and 75th percentiles, within frequency bands) were linearly interpolated using the prior and subsequent frequency band data inside the acceptable range. 6) Data were converted to a time-frequency representation via a continuous Morlet Wavelet transformation and log-transformed. The scaling size for this transformation was 3.5, which, given the 128hz sampling rate, yielded 51 bands. Six of these bands in the alpha (8-12hz) range, and these were averaged to yield an estimate of alpha power. 7) Time-frequency domain outliers (>1.5*IQR from the Tukey Hinges, as above) were then linearly interpolated. Then, time-frequency data were smoothed with a 4-second Gaussian kernel to improve the signal detection at the frequency of the design. 8) Mean continuous activity in the 8–12 Hz frequency bands were extracted and summed to yield a time-varying Alpha index and condition-related averages were generated.

### Theta burst stimulation

Stimulation was performed using a Magstim SuperRapid biphasic stimulator with a flat figure-8, remote control coil (14 cm width) with 2 T peak field strength. Stimulation percentages are expressed as a proportion of this individual unit’s maximum stimulator output (MSO). This unit can generate the theta-burst stimulation patterns at intensities of 45% MSO or below, well within range of most participant’s individual motor threshold titration as below.

Stimulation began with a determination of individualized intensity for stimulation. The figure-8 coil was held mediolaterally with the handle pointing backwards and 45° from sagittal midline as described by Brasil-Neto et al. [110]. This technique induced current perpendicular to about central sulcus. The right first dorsal interosseus muscle (FDI) was monitored with surface electromyography (5000 Hz). TMS pulses were delivered over the region of the left motor cortex in a grid at suprathreshold intensities in order to identify the location which produced the largest, most consistent motor evoked potential recorded from the FDI. Intensities at this hotspot were then lowered 1% in each stimulation. The lowest intensity stimulation that produced peak-to-peak MEP amplitudes >200 μV on at least 5 of 10 trials under conditions of gentle activation of the FDI was defined as the active motor threshold (AMT). TBS intensity was set as 80% of AMT as per Huang (2005) (cp., [111]).

Theta burst stimulation (TBS) consisted of 3 TMS pulses given at 50 Hz, with this triplet repeated every 200 ms (5 Hz). iTBS was administered as a 2 s train of TBS repeated every 10 s for a total of 190 s (600 pulses). cTBS was administered as a 40 s train of uninterrupted TBS (600 pulses).

### Identifying the TBS target site

Both cTBS and iTBS were applied to a skull area over the left dorsolateral prefrontal cortex (L-DLPFC; Brodmann area 9, approximately F3; Talairach: x = 30, y = 40,z = 26). This area was targeted based on previous functional activation studies [37]. Placement was guided by measurements of standard 10–20 system to F3 [112]. To ensure the same site was stimulated on each participant between their iTBS and cTBS session, the neuronavigation system was used just for its ability to replicate placement. Participants did not have individual fMRI scans available to guide stimulation [113]. The TMS coil was held over this location with the handle pointing backward and about 45 degrees laterally from midline.

### Procedure

Volunteers were contacted by phone to complete the screening (see above). They were scheduled on a Thursday or Friday to increase the proximity of the testing time to the weekend when they would be reporting their sexual behaviors. Given that other investigators reported difficulty documenting sufficient variability in sexual behaviors [62], and knowing sexual behaviors tend to be a bit higher on the weekends [114], weekend behaviors were targeted to increase the likelihood of greater variance in sexual behaviors. They were instructed not to orgasm either by themselves or with a partner in the 24 hours before their session. They also were instructed to abstain from any alcohol or recreational drug use in the 24 hours before their session (this is different from the inclusion criteria, which assessed whether they had heavy use of substances in the week before screening). On arrival to the laboratory, they provided written Informed Consent. The consent procedure and study protocol were approved by the Institutional Review Board at the University of California, Los Angeles.

Then, participants received instructions and practice trials for each computer task. These included the incentive delay tasks and a self-regulation task. The self-regulation task was modeled after a study by Goldin and colleagues [115] and was always presented last in the sequence of tasks. Those data are to be presented elsewhere, so are not discussed further here. Training for the incentive delay tasks included instructions and 10 practice trials. The experimenter was present for the first 3–5 practice trials to provide verbal feedback and instruction. Only monetary incentives were presented at this stage, although the participant was informed that the vibrator would be applied for the other version of this task. Tasks were completed twice: once following iTBS and once following cTBS. No sham or baseline conditions were used, because the primary purpose of this first investigation was to compare TBS types.

**Fig 2:**
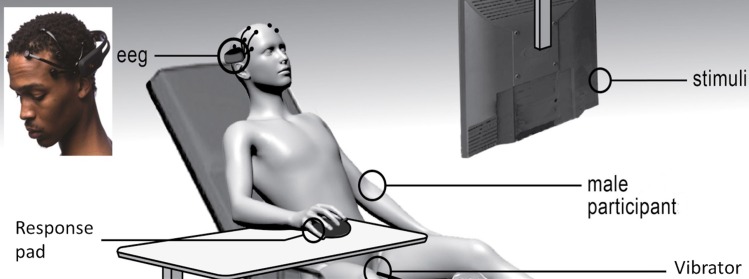
Experiment setup.

Following training, participants were led to a building next door to receive TBS. TBS order (iTBS, cTBS) was randomized. During TBS, the experimenter was not present to ensure condition blindness. Following stimulation (< 2 minutes), the participant was walked back to the private testing room (about 5 minutes). They briefly rated their emotions on a computer (cp., [116]). Next, they were seated on a massage table covered in paper and reclined for their comfort (see Fig 2). They completed the monetary incentive delay task (6 minutes). Next, the experimenter provided instructions for the placement of the vibrator, reassured the participant that the experimenter would not enter the room again without their permission, and left the private room. The participant disrobed from the waist down. The participant verbally indicated when the vibrator was in place. Then, they were instructed to set the vibrator intensity to a level that they expected would feel “good” at the longest stimulation duration of 5 seconds (see Fig 1). They controlled when the vibrator came on and turned off by key press. They verbally confirmed that the vibrator was set at the appropriate level for them before testing continued. All of this was estimated to take no more than 5 minutes even at the first test. Then, they completed the vibratory incentive delay task (6 minutes). After this task, the experimenter instructed the participant to remove the vibrator and get dressed. Together, the incentive delay tasks took about 30 minutes. Participants then completed a questionnaire concerning any pain that they experienced during or after TBS.

Two hours were required to elapse between the first TBS and the second TBS. During this period, participants completed the questionnaires on the computer (see above). At the two-hour time, they repeated TBS (counterbalanced) and MID/VID tasks. None chose to reset the intensity of the vibrator. Afterwards, participants confidentially rated two questions: “If it was free, I would be willing to go through a few of these sessions to help manage my sexual urges” and “If this works, I know someone who I think would benefit from a treatment like this” on a scale from 1 (Strongly agree) to 7 (Strongly disagree). They were then provided with instructions for completing the Weekend Activities questionnaire (see above), provided an opportunity to ask questions, received $100 cash, and left. The entire protocol took 4 hours. The following Monday they received automated reminders to complete their Weekend Activities Questionnaire. They received $50 for completing the questionnaire. Most (n = 18) elected to receive payment in cash returning to the laboratory, whereas others (n = 2) provided identifying information to receive payment by mailed check.

### Data analyses

Two analyses were planned. First, the effects of TBS type on reward responsiveness were tested. Specifically, average alpha band activity across electrodes was predicted using a two-factor, within-participant ANOVA by TBS type (iTBS, cTBS) and reward phase (anticipation, receipt). This was done separately for reward phase (anticipation, receipt) and reward type (money, sex).

Second, the ability of EEG responses to sexual rewards in the lab were used to predict real-world sexual behaviors in the weekend following stimulation. Given that others have found insufficient variability in the number of partners, which was also true in this study, total orgasm count was used as a proxy dependent variable. Specifically, alpha activity to the sexual rewards (identified in the first analysis) at all sites was used to predict orgasm count. A cumulative link model (CLM) was used (R library ordinal; Christensen, 2015), which allows prediction of each level of orgasm response. This approach predicts the probability that a response (alpha) will fall in a particular category (orgasm count) or below, hence “cumulative”. CLM offers several advantages for these data. CLM allows non-linear response patterns. CLM relies on a single weight variable, making it more parsimonious than alternative models. Finally, CLM accounts for the likelihood that orgasm count is conservatively characterized as ordinal. The order of the brain stimulation received was included as a predictor to control for the unlikely possibility that the brain stimulation type received most recently might influence orgasm count over the coming weekend. TBS type also was initially included as a predictor to ensure that it was reasonable to collapse across TBS sessions to power the analyses, as no difference was expected in the predictive utility of alpha for orgasm count. As TBS type did not interact with alpha to predict orgasm count, data are collapsed across TBS sessions and not discussed further. EEG site was included as a predictor to ensure specific site outliers were not driving observed effects.

### Time course analysis

Contrasts on alpha EEG at each electrode were examined via statistical tests at each point along the wavelet-derived reactivity waveforms. To control type 1 error for this large number of tests, Guthrie and Buchwald’s [117] technique was used. Briefly, this technique involves using Monte-Carlo simulations to estimate the number of consecutive significant differences long enough to be judged to not have occurred by chance with *p* < .05 given the temporal autocorrelation of the data. Given the sample autocorrelation of 0.997, 129 contiguous sample-by-sample tests (approximately 1 second), each significant at *p*<0.1 were considered replications at *p*<0.05.

## Results

All participants completed all aspects of the protocol. One male participant denied being sleepy during the tasks following iTBS, but during that period showed EEG largely devoid of eyeblinks, characterized by slow wave activity, and failed to respond on most reward trials. Conservatively, his EEG data were excluded from analyses as he could have been asleep during this time. Data also were lost from 3 iTBS trials due to experimenter error. One participant reported tension-type head pain (reaching 6 of 11 pain rating) following cTBS stimulation. This participant received an over-the-counter analgesic and reported that the pain had resolved within 20 minutes. Two participants had motor thresholds in excess of the MagStim capability. These were lowered by 3% and 10% of the measured threshold. In general, the AMT mean was 51.4 and standard deviation was 7.7.

### Vibrator intensity

Participants chose to set the vibrator intensity from 2 to 9 with *M*(*SD*) = 5.2(2.5). Given that the mid-point of intensity was 5, this is consistent with individuals using the full range of the available settings for the device. No one chose to leave it in the off (“1”) position.

### Alpha response differed by reward condition and task

**Fig 3:**
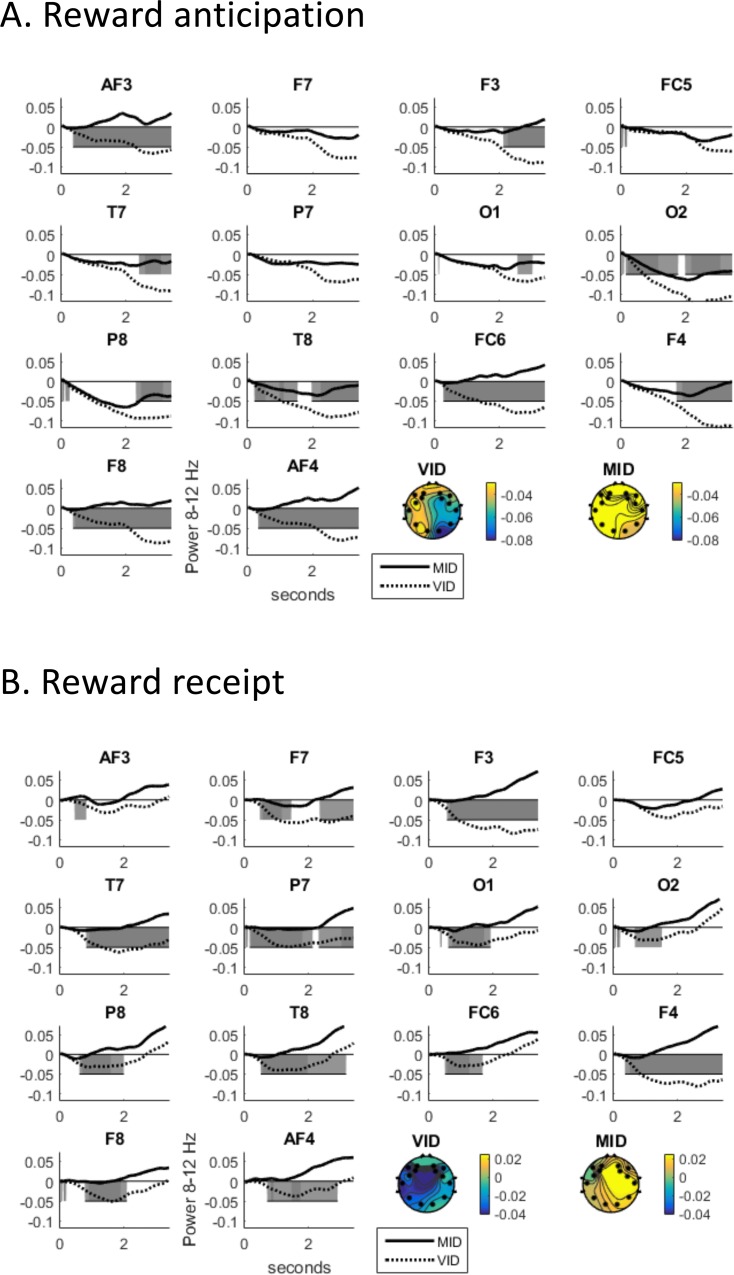
Alpha in response to reward for MID and VID during (A) Anticipation and (B) Receipt (both TBS conditions included).

**Fig 4:**
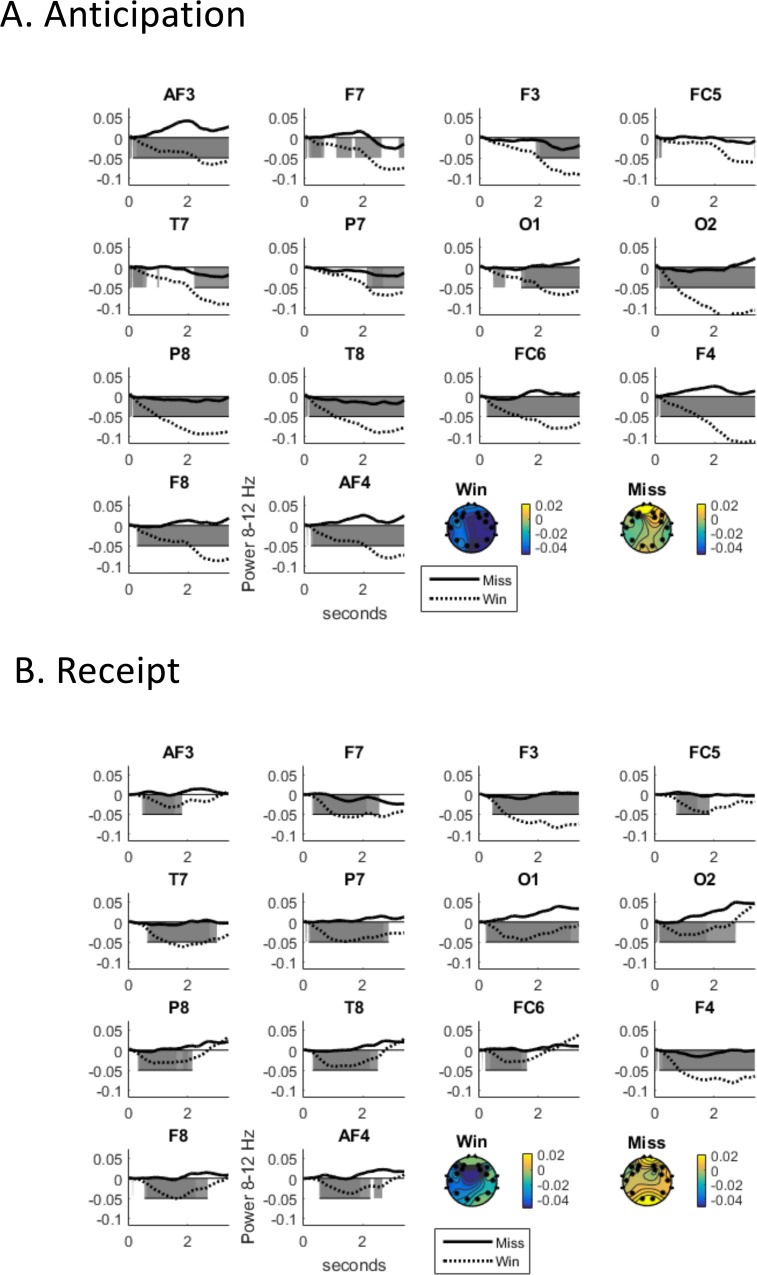
Alpha in response to sexual reward (win) versus non-reward (miss) (A) Anticipation and (B) Receipt (both TBS conditions included).

VID rewards suppressed alpha more than MID rewards for both anticipation and receipt phases (see Fig 3). Alpha EEG differed (see Table 1 and Fig 4 for which electrodes this was significant or merely consistent) across the scalp in response to wins and misses for sexual rewards throughout the time-course of the anticipatory (post-response, pre-vibratory stimulus) interval as well as the receipt interval (during vibratory stimulus). Along with previous publications showing the utility of alpha EEG for sexual rewards [49], this supports the chosen EEG metric of alpha suppression as one index of reward responsivity in VID. Since alpha did not reliably differentiate receipt for wins and misses in the MID version, EEG alpha to rewards in the VID task were used for remaining comparisons.

**Table 1:** Significant difference by trial type (win/miss) at each electrode for sexual rewards (anticipation/receipt)

Sensor	t	D(s)	d
Anticipation			
AF3	-3.1	-0.06	-0.52
F7			
F3	-2.36	-0.06	-0.39
FC5			
T7	-1.94	-0.06	-0.32
P7	-1.98	-0.05	-0.33
O1	-3.07	-0.06	-0.51
O2	-4.4	-0.08	-0.73
P8	-3.52	-0.06	-0.59
T8	-3.55	-0.05	-0.59
FC6	-4.03	-0.06	-0.67
F4	-3.84	-0.08	-0.64
F8	-3.48	-0.06	-0.58
AF4	-3.45	-0.06	-0.58
Receipt			
AF3	-2.27	0.03	-0.38
F7	-2.71	0.01	-0.45
F3	-3.81	-0.06	-0.64
FC5	-2.07	0.05	-0.34
T7	-2.64	0.01	-0.44
P7	-3.17	-0.04	-0.53
O1	-3.4	-0.05	-0.57
O2	-2.3	0.03	-0.38
P8	-2.42	0.02	-0.4
T8	-3.28	-0.03	-0.55
FC6	-2.74	0.01	-0.46
F4	-3.08	-0.05	-0.51
F8	-2.87	0.01	-0.48
AF4	-2.32	0.03	-0.39

### EEG predicted sexual behaviors

**Fig 5:**
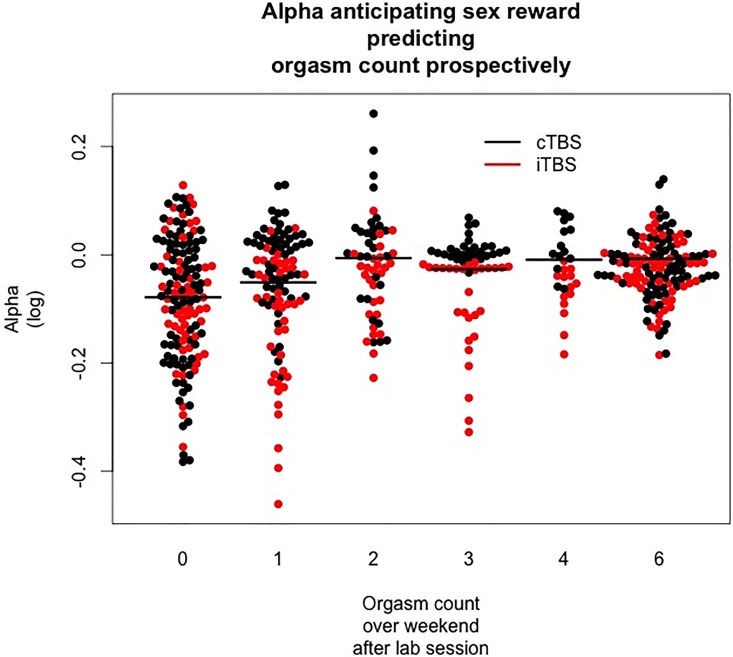
Prediction of orgasm count over the coming Friday, Saturday, and Sunday by EEG alpha in anticipation of sex reward (both brain stimulation conditions included). The plot reflects the ordinal assumptions of conditional logit models (CLM). CLM do not require interval spacing, only that each “level of orgasm” be ordered For example, no value of 5 exists because no one reported 5 orgasms, and each logit is calculated only relative to the next value in the ordered list of orgasm counts.

Participants varied considerably in the number of orgasms that they experienced over the weekend (see Table 2), making this variable suitable for analysis. The CLM model generally fit the data (log likelihood = 823, AIC = 1662) after seven fit iterations. There was a main effect of Alpha (*z* = 5.8, *CI* = 3.2 to 25.8, *p* = .02; see Fig 5), but no main effect of stimulation order (*z* = .4, *p* = .06) or electrode (*z* = -0.14, *p* = .89) nor any interaction (order by electrode, order by alpha, electrode by alpha, or order by electrode by alpha). Alpha level coefficients showed that those who had stronger alpha suppression anticipating sexual reinforcement also reported fewer orgasms (z-coefficients 0|1 = -1.3, 1|2 = .3, 2|3 = 1.2, 3|4 = 2.1, 4|6 = 2.6).

**Table 2:** Sample characteristics.

Variable	Mean	Standard deviation
Age	34.6	10.9
Lifetime intercourse partners	22.5^a^	38.5
Behavioral Inhibition^b^	17.0	3.0
Behavioral Activation^c^	19.6	8.9
Sensation seeking		
Thrill And Adventure Seeking	6.3	2.8
Disinhibition	5.6	2.4
Boredom Susceptibility	2.5	1.7
Experience Seeking	5.0	2.3
Sensitivity to		
Rewards	33.8	4.3
Punishments	41.1	4.3
Weekend activities		
Sexual partners	0.8	1.1
Orgasms	2.4	2.5
Usual risk^d^	3.5	1.1
	N	%
Women	5	25
Relationship		
Monogamous	5	25
Non-monogamous	6	30
Not in relationship	9	45
Ethnicity		
Asian	1	5
African-American	7	35
Hispanic	6	30
White	4	20
Other	2	10

^a^Five people reported > 100 partners, so median is reported for this variable to decrease the influence of skew

^b^Range 7 to 32

^c^Range 13 to 52

^d^Participants were asked whether their sexual activities over the weekend reflected their usual sexual activities with Range 1 - “Much more risky sexually than usual” to 5 - “Much less risky sexually than usual”

### Alpha response to rewards differed by TBS type

**Fig 6:**
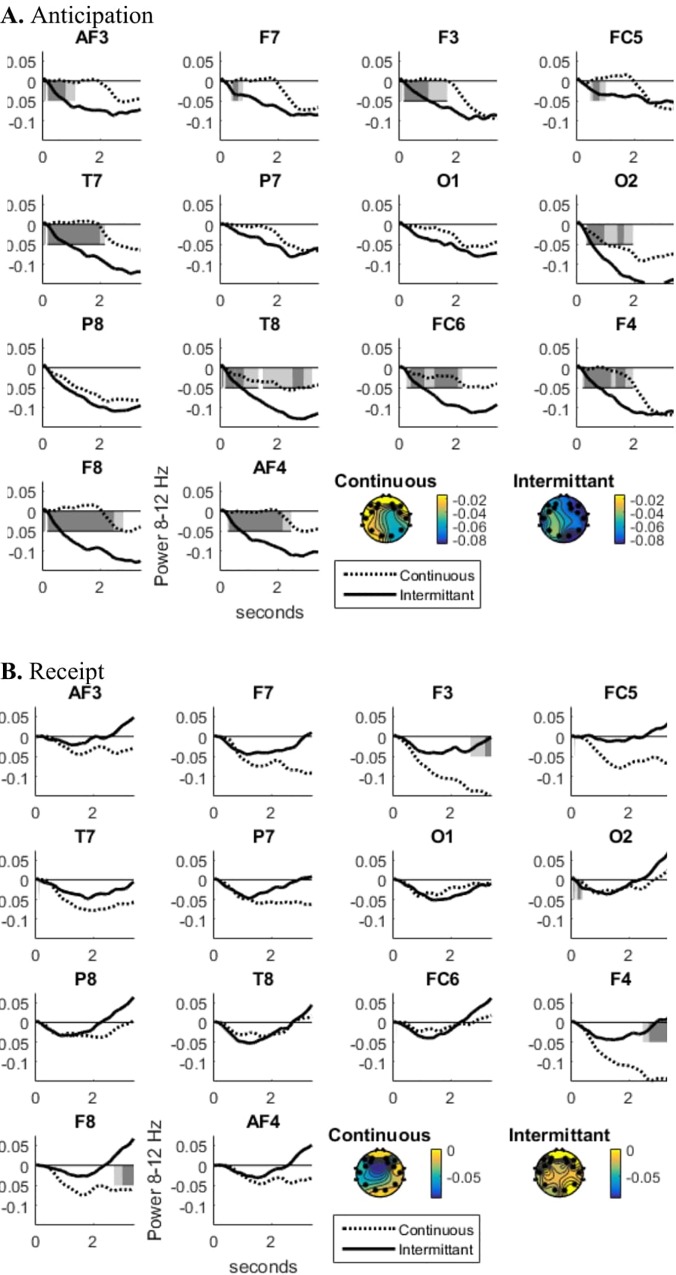
Alpha during vibratory incentive delay to (A) anticipation and (B) receipt.

First averaging alpha across electrodes, a main effect of TBS type predicted alpha power in the vibratory incentive delay task, *F*(1,64) = 6.1, *p* = .02. Specifically, alpha power was lower following iTBS (*M(SD*) = -.05(.08)) as compared to cTBS (*M(SD*) = -.03(.09)) for both the anticipation and receipt of vibrator rewards. There was no effect of TBS type on monetary rewards (anticipation or receipt). The time series of the TBS effect across each electrode supports this averaged result (see Fig 6). That is, most electrodes show a suppression of alpha specific to iTBS anticipating the sex reward and, to a lesser extent, receiving the sex reward. Effect sizes at electrodes showing significant differences varied from *d* = .78 to *d* = 1.16 in temporal windows of significant differences (see Table 3). See S1 Fig for alpha response by each predictor level.

**Table 3:** Difference by stimulation type (cTBS/iTBS) at each electrode for anticipation of sex reward.

Sensor	t	D(s)	d^1^
AF3	-1.8	-0.06(0.09)	-.6
F7	-1.34	-0.04(0.08)	-.45
F3	-2.35*	0.05(0.07)	-.79
FC5	-2.32*	-0.06(0.08)	-.78
T7	-2.52*	-0.07(0.08)	-0.85
P7	-2.64*	-0.06(0.07)	-0.88
O1	0.02	0.00(0.07)	.01
O2	-0.74	-0.02(0.08)	-.25
P8	-1.6	-0.05(0.08)	-0.54
T8	-1.25	-0.03(0.07)	-0.42
FC6	-1.34	-0.03(0.08)	-0.45
F4	-3.44**	-0.09(0.08)	-1.16
F8	-1.88	-0.05(0.09)	-0.63
AF4	-1.33	-0.04(0.09)	-0.45

* *p* < .05

** *p* < .01

^1^Effect size for dependent test, Averaged across electrode sites $\eta_{p}^{2}$ = .01.

### Postexperimental questionnaire

Response options ranged from 1 (“Agree strongly”) to 7 (“Disagree strongly”). Ten participants (50%) reported that they were open to receiving more TBS sessions to assist them managing their sexual urges (rated 4 or below). Fourteen participants (70%) felt they knew someone whom they thought would benefit from TBS to dampen sexual responsiveness if it proves effective (rated 4 or below).

## Discussion

In a sample of 20 higher-sexual risk men and women, EEG was recorded while they completed reward tasks. They received theta burst brain stimulation designed to increase or decrease activity in the L-DLPFC. After the session, they reported their sexual behaviors over the next weekend. Alpha was reliably lower in response to anticipation and receipt of sexual rewards, compared to non-reward and compared to monetary reward. Decreased alpha to the anticipation of sexual rewards predicted orgasm count in the coming weekend. Furthermore, iTBS was associated with lower alpha to sexual rewards (anticipation and receipt) as compared to cTBS. These data suggest that brain stimulation can be used to modulate responsiveness to reward features that are associated with health-risk behaviors. Further, these results alpha EEG change to sexual rewards as an experimental therapeutics target. Specifically, alpha appears appropriate for target identification (lower with sexual reward), to target as a risk biomarker (predicts sexual behavior), and a malleable mechanism for problems of reward (altered by TBS).

Prospective data supported the external validity of EEG alpha suppression to primary rewards in the laboratory. Brain responses have been linked to the number of past sexual partners [6] and future sexual desire levels [62]. These data provide additional validity supporting the use of neural responses to sex rewards as a potential biomarker for real-world sexual behaviors. The direction of the effect suggests that those who were more sexually responsive (greater alpha suppression anticipating sexual rewards) in the laboratory experienced fewer sexual rewards (orgasms) in the following weekend. This seems counterintuitive, because greater sexual motivation in the laboratory should lead to more sexually motivated behaviors. Interpreting alpha as a general measure of activation, impulsivity literature may aid understanding of this effect. For example, those with higher P300 to visual oddballs in a laboratory task reported less impulsivity (Russo, Pascalis, Varriale & Barratt, 2008). This may reflect that the lesser activity in ventral prefrontal cortex associated with impulsivity that EEG may be more sensitive to than deeper structures (Brown, Manuck, Flory, & Hariri, 2006). Components of evoked response potentials have proven more specifically sensitive to rewards (e.g., Martin & Potts, 2004), where alpha activity is probably better understood as an index of idling or arousal. To more strongly interpret this effect, it will be helpful to include physiological indices more specific to reward or bring participants back for reassessment to characterize shifts in responsiveness.

These data suggest that sexual stimulation offers a unique target above and beyond secondary reward stimuli. The MID version of the task did not reliably differentiate wins from misses in alpha EEG and generally was less effective at suppressing alpha compared to the VID. Others have documented EEG modulation in theta and beta ranges [118]. Alpha may not best capture MID modulations. This could reflect a difference in primary and secondary reinforcement types, but that was not explicitly tested. A second possibility is that the MID and VID were always conducted in the same order. This allowed participants to only have their genitalia exposed to the vibrator for the minimum amount of time necessary. One argument against this order problem is that the TBS effects should have faded less for the earlier (MID) task. However, it also is possible that reward states were somehow additive such that having just completed the MID influenced responses to the VID. A third possibility is that this sample was especially sensitive to sexual rewards. Since individual differences in reward responsiveness predict reward learning in the MID [119], it seems reasonable that this sample simply might be less responsive to secondary money rewards. Relatedly, TBS differences might be limited to individuals with reward hypersensitivity. The applicability to sexual reward hyposensitivity is suggested, but not directly supported. Another possibility is that this could reflect differences in general arousal, differences which may just happen to be greater in sexual stimuli (see Supplement).

cTBS appeared to decrease responsiveness to the anticipation of primary rewards relative to iTBS. Since cTBS both decreases the activity of DLPFC and decreases the activity of dopamine in deeper brain structures important to reward (reviewed above), its mechanism remains unclear. In support of the role of targeting the DLPFC, decreased activity in this structure appears to aid in the maintenance of a flaccid state of the penis [120]. Furthermore, iTBS of L-DLPFC has antidepressant effects [121], which appears consistent with iTBS enhancing the effects of sexual reward anticipation. TMS effects have been described as either directly affecting task-relevant cortical areas or inhibiting task irrelevant networks as new response patterns are learned [122].

A primary limitation of this study is the lack of a TBS sham. Since this was the first time TMS/TBS has been used to modulate sexual feelings or behaviors, we felt it was important to contrast excitatory and inhibitory stimulation types before a next stage of study than a sham or baseline control. These data provide some confidence that (1) TBS modifies responsiveness to primary rewards and (2) the type of TBS to pursue depending on the goals of enhancing or reducing responsiveness. Specifically, studies aimed at increasing the anticipation of sexual rewards should choose iTBS, whereas studies aimed at decreasing the anticipation of primary rewards should choose cTBS.

Other study limitations exist. L-DLPFC was targeted without the benefit of stereotaxic MRI guidance. Individuals vary in the location of brain areas with respect to external scalp landmarks [123]. This method of site identification has been critiqued [124]. Inter-expert variation is a main source of variability in this method [125], which was eliminated here by the use of a single expert technician (C.B.). Others are continuing to develop methods to increase the reliability of F3 identification using landmark approaches [126]. Nevertheless, it will be desirable to verify this pattern of results with anatomically-targeted stimulation in the future. Third, the investigation was limited to alpha band activity. One other study of smoking urges examined delta patterns based on existing smoking literature [127]. The current study specifically examined reward responsiveness. Since alpha activity has been extensively tied to depression and anhedonia and sexual stimulation (see above), alpha was a reasonable target. However, it is possible that this focus may have overlooked other important changes in band activity. While the protocol maximized the time between stimulation types informed by studies about the length of stimulation effects, it is possible carryover caused some interference. Counterbalancing (cTBS and iTBS) ensured that this was not systematic, but merely having had some stimulation could cause some interaction with our tests. Finally, actual money was not handed to the participant following the money rewards, which may have created an additional difference between the money and sex rewards. Studies of real-money versus fake money reinforcers suggest little difference in results between the two approaches [128], but providing real money would have increased the similarity with the sexual reward.

This study provides initial support for a mechanistic intervention to alter a neural index related to real-world sexual behaviors. More broadly, these data support that TBS is useful to change responsiveness to rewards. Studies including secondary and primary reinforcements remain rare (e.g., [129]). When investigated, deficits responding to primary sexual rewards better characterize reward differences in problem gamblers [130], and responsiveness to sexual rewards is specifically recovered in successful depression treatment [131]. Strong primary rewards, such as sexual rewards, may test the ability of the reward system to respond in completely different ways from traditional secondary rewards. This study specifically altered processing of primary rewards, which may offer new therapeutic targets in TBS interventions.

## Supporting Information

S1 Fig Alpha in response to reward for MID and VID during (A,C) Anticipation and (B,D) Receipt. Fig 2. Alpha in response to reward for MID and VID during (A,C) Anticipation and (B,D) Receipt.
(DOCX)
