# Supplementary material for: EEG to Primary Rewards: Predictive Utility and Malleability by Brain Stimulation
Source: PLoS One. 2016 Nov 30;11(11):e0165646. doi: 10.1371/journal.pone.0165646 (PMC5130195; doi:10.1371/journal.pone.0165646)

Supplement

*Supplement Figure 1.* Alpha in response to reward for MID and VID during (A,C) Anticipation and (B,D) Receipt

Intermittent

1.
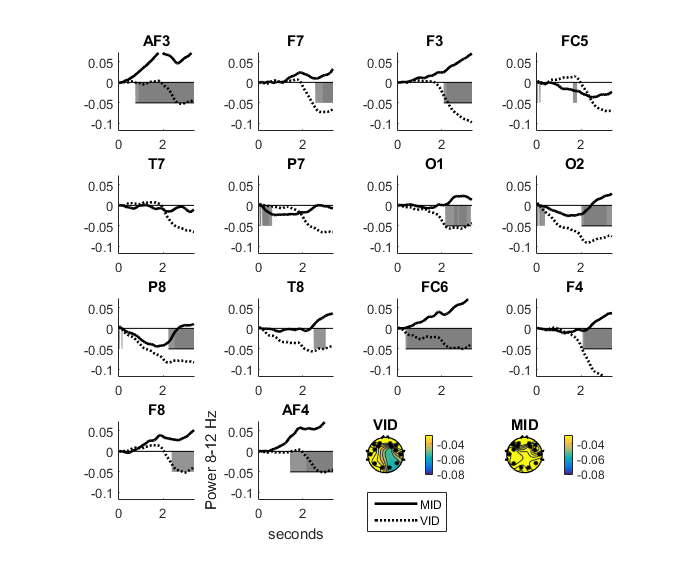
Reward anticipation
2.
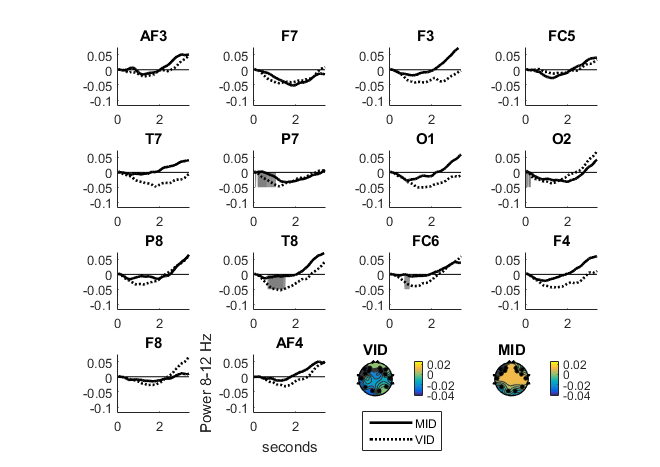
Reward receipt

Continuous

1. Reward anticipation


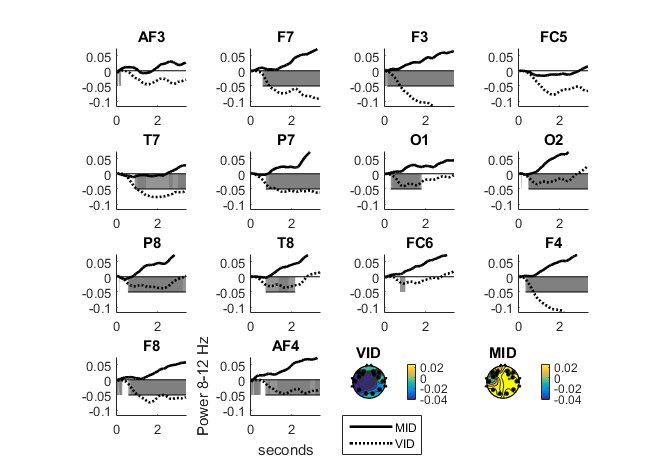


1. Reward receipt


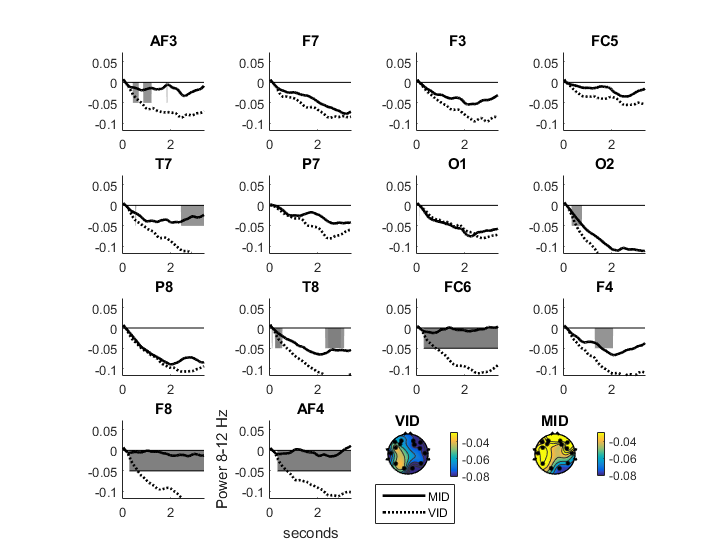


*Supplement Figure 2.* Alpha in response to reward for MID and VID during (A,C) Anticipation and (B,D) Receipt

Intermittent

1. Anticipation


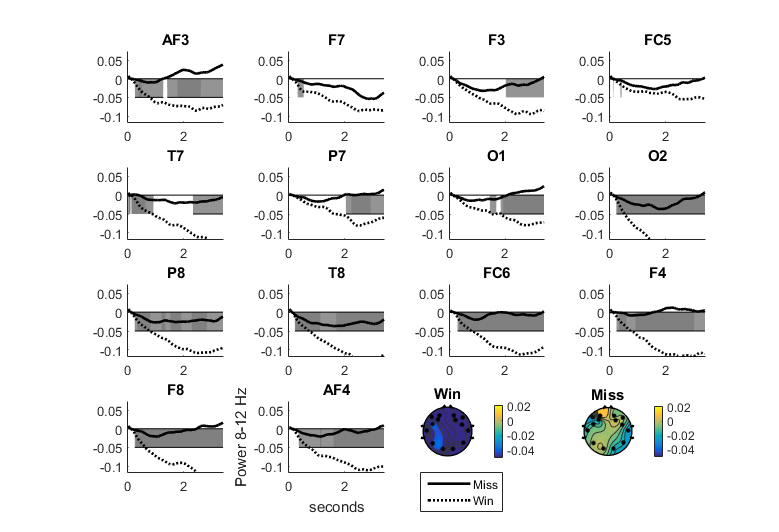


1. Receipt


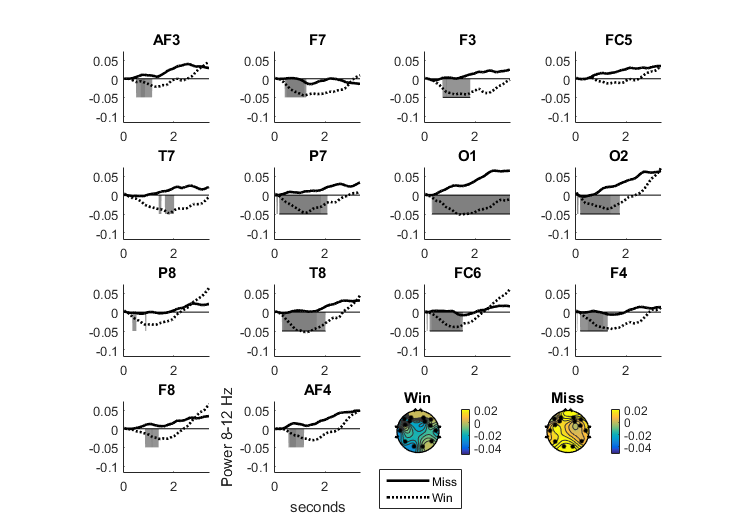


Continuous

1. Anticipation


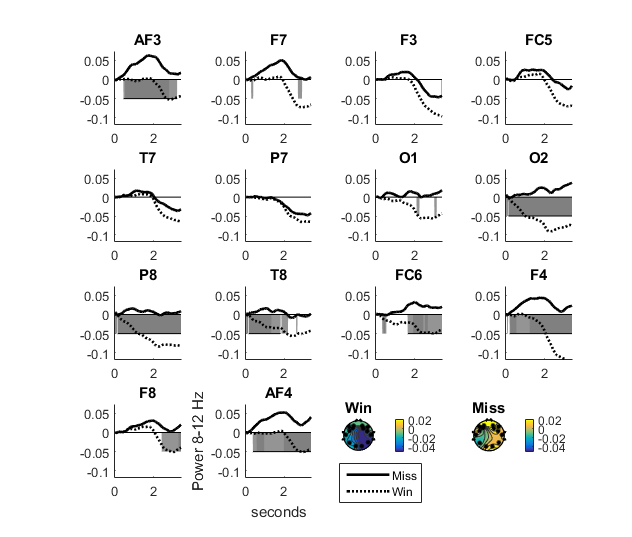


1. Receipt


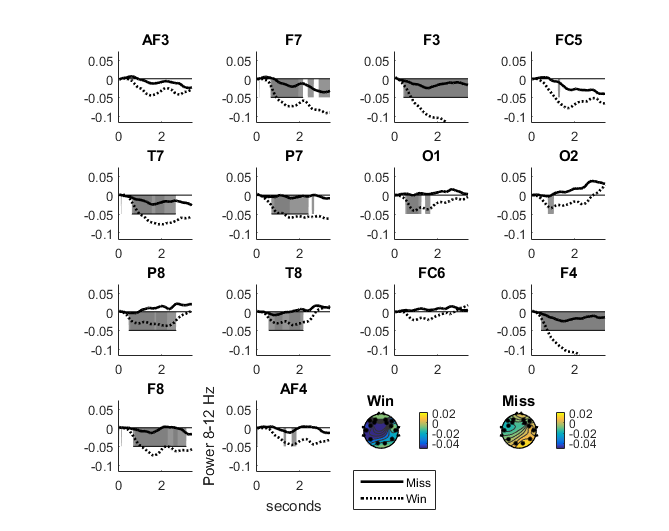

Supplement: S1 Fig — Alpha in response to reward for MID and VID during (A,C) Anticipation and (B,D) Receipt. Fig 2. Alpha in response to reward for MID and VID during (A,C) Anticipation and (B,D) Receipt. (DOCX) [file pone.0165646.s001.docx]
